# Fatty acid binding proteins 4 and 5 in overweight prepubertal boys: effect of nutritional counselling and supplementation with an encapsulated fruit and vegetable juice concentrate

**DOI:** 10.1017/jns.2015.29

**Published:** 2015-12-02

**Authors:** Jose A. Canas, L. Damaso, J. Hossain, P. Babu Balagopal

**Affiliations:** 1 Pediatric Endocrinology and Metabolism, Nemours Children's Specialty Care, Jacksonville, FL 32207, USA; 2 Bioinformatics Core Facility, Nemours Children's Specialty Care, Jacksonville, FL 32207, USA; 3 Biomedical Research, Nemours Children's Specialty Care, Jacksonville, FL 32207, USA

**Keywords:** Obesity, Children, Fatty acid binding proteins, β-Carotene, AIR, acute insulin response, FABP, fatty acid binding protein, FVJC, fruit and vegetable juice concentrate, NC, nutritional counselling, OW, overweight

## Abstract

Elevated fatty acid binding proteins (FABP) may play a role in obesity and co-morbidities. The role of nutritional interventions in modulating these levels remains unclear. The aim of this *post hoc* study was to determine the effect of overweight (OW) on FABP4 and FABP5 in boys in relation to indices of adiposity, insulin resistance and inflammation, and to investigate the effects of a 6-month supplementation with an encapsulated fruit and vegetable juice concentrate (FVJC) plus nutritional counselling (NC) on FABP levels. A *post hoc* analysis of a double-blind, randomised, placebo-controlled study of children recruited from the general paediatric population was performed. A total of thirty age-matched prepubertal boys (nine lean and twenty-one OW; aged 6–10 years) were studied. Patients received NC by a registered dietitian and were randomised to FVJC or placebo capsules for 6 months. FABP4, FABP5, glucose, insulin, homeostasis model assessment-insulin resistance (HOMA-IR), glucose-induced acute insulin response (AIR), lipid-corrected β-carotene (LCβC), adiponectin, leptin, high-sensitivity C-reactive protein (hs-CRP), IL-6 and body composition by dual-energy X-ray absorptiometry were determined before and after the intervention. FABP were higher (*P* < 0·01) in the OW *v.* lean boys and correlated directly with HOMA-IR, abdominal fat mass (AFM), hs-CRP, IL-6, and LCβC (*P* < 0·05 for all). FABP4 was associated with adiponectin and AIR (*P* < 0·05). FVJC plus NC reduced FABP4, HOMA-IR and AFM (*P* *<* 0·05 for all) but not FABP5. OW boys showed elevated FABP4 and FABP5, but only FABP4 was lowered by the FVJC supplement.

Childhood nutrition plays a significant role in the management of obesity and related co-morbidities such as CVD and type 2 diabetes mellitus. Alterations and/or deficiencies in lipophilic micronutrients may contribute to the excess accumulation of intra-abdominal adipose tissue and dysregulation of carbohydrate and lipid metabolism^(^^1^^,^^2^^)^. Fatty acid binding proteins (FABP) are members of a highly conserved tissue-specific family of cytosolic lipid chaperones expressed in adipocytes and macrophages^(^^3^^)^ that may contribute to the regulation of energy metabolism, insulin resistance and inflammation by activating nuclear receptors such as retinoic acid receptors and PPAR^(^^4^^)^. FABP are also implicated in the cellular uptake and transport of lipophilic micronutrients that integrate metabolic and inflammatory responses involved in obesity-related diseases^(^^5^^,^^6^^)^. In animal models, FABP4 deficiency protects against the development of insulin resistance, inflammation, diabetes and atherosclerosis in both genetic and dietary forms of obesity^(^^7^^,^^8^^)^. In adult humans, decreased FABP4 expression is associated with lower TAG and reduced risk for CVD and type 2 diabetes mellitus^(^^9^^)^. While elevated levels of FABP4 have also been reported both in obese children and adults, its relationship with obesity-related risk factors for CVD remains less clear^(^^10^^–^^16^^)^. Children with lower FABP4 concentrations who consume high-fat diets do not show a concomitant increase in fasting insulin or high-sensitivity C-reactive protein, whereas those with higher FABP4 concentrations show marked increases in these measures, which correlate with the intake of saturated fat^(^^13^^)^. In adults, FABP4 has been shown to correlate directly with the glucose-induced acute insulin response (AIR), and is postulated to play an integral role in coupling β-cell function with adiposity^(^^17^^)^. A few studies have also reported the reversal of the elevated FABP4 levels after weight reduction in children^(^^12^^,^^18^^)^. Because FABP play important roles and contribute to obesity-related co-morbidities, developing nutritional strategies to reduce their levels is important.

The primary objectives of this *post hoc* analysis performed on a double-blind placebo-controlled study was to determine the effect of overweight (OW) on FABP4 and FABP5 and the effect of nutritional counselling (NC) and supplementation with fruit and vegetable juice concentrate (FVJC) on these levels in lean and OW boys.

## Experimental methods

### Trial procedures

This study was conducted in accordance with the guidelines laid down in the Declaration of Helsinki, and all procedures involving human subjects/patients were approved by the Institutional Review Committee at Wolfson Children's Hospital, Jacksonville, FL. Written parental informed consent and child's assent were obtained for all participants upon enrolment in the study. Subjects received a modest monetary compensation for their participation in the study. The study was registered at http://www.clinicaltrials.gov (NCT00842543).

### Participants

Detailed description of the subjects as well as data on certain cardiometabolic risk factors in the same subjects have been previously reported^(^^19^^)^. Briefly, this was a *post hoc* analysis performed on a total of thirty-nine prepubertal boys (age 6–10 years) enrolled, thirty completers (nine lean with BMI ≤ 85th percentile and twenty-one OW with BMI > 85th percentile) and nine dropouts (due to fear of the second blood draw).

Subjects with a history of chronic illness or chronic medications were excluded from the study. Those with illness or bone fracture within 2 weeks of their blood draw were also excluded. The participants were instructed not to consume any medications, including vitamins, herbal remedies or anti-inflammatory drugs within 30 d of the anticipated blood draw.

### Randomisation

The intent-to-treat principle was applied to thirty-nine subjects who were randomised utilising a randomisation scheme generated by the website Randomization.com (http://www.randomization.com) and who received either active supplement or identical placebo capsules provided by the manufacturer (Juice Plus+®; NSA, LLC) in conjunction with 6 months of NC.

### Design

The detailed study protocol and design have been previously described^(^^19^^)^. A modified rapid intravenous glucose tolerance test was performed as previously described^(^^20^^)^. All subjects received a 60-min NC session with a registered dietitian. Instructions emphasised to limit saturated fats to *<*7 % of total energy intake, with a minimum of five servings of fruits and vegetables per d, and moderate daily activity was encouraged. Instructions were re-emphasised after 3 months. Subjects were instructed to take one study capsule with breakfast and dinner. Two active capsules of the supplement provide approximately 3·75 mg β-carotene, 117 mg of vitamin C, 22·5 IU of vitamin E (23 mg α-tocopherol equivalents), 210 µg folate, 30 mg Ca and 21 kJ per d. The orchard concentrate consists of 850 mg of dried powder blend of juice and pulp (52 %) with apple, orange, pineapple, cranberry, peach, acerola cherry and papaya in varying proportions, in addition to beetroot powder, date fibre and prune fibre. The garden blend contains 750 mg of dried powder blend of juice and pulp (60 %) of carrot, parsley, beet, kale, broccoli, cabbage, tomato and spinach, in varying proportions, as well as sugar beet fibre, garlic powder, oat bran fibre and rice bran. Moreover, each powder was enriched with vitamins (vitamin C, vitamin E and folic acid) and carotenoids (β-carotene). These supplements are commercial products that have been recently chemically characterised by an independent group. Within the orchard blend, twenty-eight compounds with a predominance of flavonols have been identified. A total of twenty-five compounds, belonging to different phenolic classes have been identified within the garden blend^(^^21^^)^.

### Assays

Blood was collected after 10 h of fasting and carefully processed under orange lights immediately after collection; serum and plasma were aliquoted and frozen in opaque tubes at −80°C until analysis. FABP4 (Cayman Chemical; interassay CV 3·9 %) and FABP5 (BioVendor; interassay CV 5·8 %) were measured by ELISA. The following measurements were also made as previously described^(^^19^^)^. β-Carotene was measured by HPLC with photodiode array detection, glucose was measured using an Analox GM7 Glucose Analyser (Analox Instruments; interassay CV < 5 %) and insulin was measured by a commercially available RIA kit (EMD Millipore Life Sciences; interassay CV < 10 %). Total cholesterol, TAG and HDL-cholesterol concentrations were measured using colorimetric assays (Beckman DxC 800 Analyzer; Beckman Coulter, Inc.; interassay CV < 2·6, <2, and <3 %, respectively), IL-6 by specific ELISA (R&D Systems; interassay CV 7·8 %), high-sensitivity C-reactive protein by immuno-nephelometry (Siemens Healthcare Diagnostics; interassay CV < 5 %) and adiponectin and leptin by RIA (EMD Millipore Life Sciences; interassay CV 8·5 and 4·5 %, respectively).

### Calculations

The homeostasis model assessment-insulin resistance (HOMA-IR) was calculated using the following formula: fasting glucose (mmol/l) × fasting insulin (μU/ml)/22·5. AIR was defined as the mean incremental rise in plasma insulin at 3 and 5 min after a rapid intravenous load of glucose. To adjust AIR for the effects of insulin sensitivity, a glucose disposal index was calculated as log_10_ (AIR × fasting glucose concentration/fasting insulin concentration) in a manner similar to the correction of AIR using the insulin sensitivity index that is used in the minimal model intravenous glucose tolerance test^(^^22^^)^.

β-Carotene concentrations are closely correlated with major lipid distribution and were corrected for lipid status (lipid-corrected β-carotene) by dividing by the sum of total cholesterol and TAG expressed in mmol/l^(^^23^^)^. The leptin:adiponectin ratio was also calculated.

### Statistical analyses

The intention-to-treat principle was applied to all patients included in the primary analysis. In the *post hoc* analysis, quantitative variables are presented using either mean with standard deviation or in the case of substantially skewed distribution, median and interquartile range. Categorical variables are presented using frequencies and percentages. The two-sample *t* test or a non-parametric Mann–Whitney *U* test, whichever was appropriate, was used to compare quantitative variables. The Wilcoxon signed-rank test was used for paired sample comparisons of non-parametric variables.

A multivariate ANOVA for repeated measures was performed to compare the mean changes from baseline at the 3-month and 6-month visits for the variables of interest. The least squared means with standard errors and *P* values for both lean and obese subjects are presented at 3 and 6 months in Table 2. Both models were adjusted for baseline values of the corresponding variable and the percentage change in weight. Associations between FABP4 and FABP5 with other variables were examined with unadjusted univariate analysis followed by multivariate linear regression analysis, adjusting for potential confounders to determine independent predictors of plasma FABP4 and FABP5. All tests were two-tailed at the level of significance of ≤0·05. The statistical software SPSS version 22.0 (SPSS) was used for analyses.

## Results

### Participant characteristics

The clinical and biochemical characteristics of the study participants by BMI (Table 1) and treatment group are presented in Table 2. Although subject characteristics and some biological factors have been published previously^(^^19^^)^, for clarity of discussion, we have included some of the data pertinent to the present study along with new data on FABP4 and FABP5.

**Table 1.** Baseline clinical and biochemical characteristics of study subjects by weight group (Mean values and standard deviations; medians and interquartile ranges (IQR))

	Lean (*n* 13)		Overweight (*n* 26)		P*
	Median	IQR	Median	IQR	
Age (years)					NS
Mean	9·36		9·04		
sd	1·31		1·43		
BMI *Z*-score					<0·05
Mean	−0·08		2·14		
sd	1·43		0·47		
Abdominal fat mass (kg)					<0·05
Mean	1·249		4·144		
sd	0·391		0·141		
LCβC (μmol/l)	0·055	0·038–0·093	0·035	0·023–0·066	0·04
FABP4 (ng/ml)†	9·92	8·5–11·4	30·67	26·7–34·6	<0·001
FABP5 (ng/ml)†	3·33	2·8–3·9	6·76	5·7–7·9	<0·001
TAG (mmol/l)	0·52	0·37–0·75	0·96	0·61–1·20	0·004
HOMA-IR	1·34	0·8–1·82	3·68	1·98–4·01	<0·001
AIR	345	206–597	687	402–1,014	0·014
GDI					NS
Mean	1·67		1·50		
sd	0·30		0·25		
hs-CRP (mg/l)	0·36	0·16–0·39	0·93	0·38–0·93	0·01
IL-6 (pg/ml)	0·69	0·64–1·07	1·35	0·92–2·3	0·018
Leptin (ng/ml)	3·76	2·5–4·8	21·3	10·6–26·5	<0·001
Adiponectin (μg/ml)	14·7	10·8–20·9	10·7	7·6–12·4	0·04
Leptin:adiponectin ratio	0·23	0·19–0·31	1·85	1·02–2·67	<0·001

LCβC, lipid-corrected β-carotene; FABP, fatty acid binding protein; HOMA-IR, homeostasis model assessment-insulin resistance; AIR, acute insulin response; GDI, glucose disposal index; hs-CRP, high-sensitivity C-reactive protein.

* *P* values represent differences between groups as determined by the independent two-sample *t* test or Mann–Whitney test for continuous variables.

† FABP4 and FABP5 data have not been previously reported^(^^19^^)^.

**Table 2.** Mixed-model analysis of the changes in concentrations between fruit and vegetable juice concentrate (FVJC) and placebo in the overweight group* (Least squared means (LS means) with their standard errors)

	FVJC (*n* 11)				Placebo (*n* 10)						
	0 months		6 months		0 months		6 months		Mean difference		
	LS mean	se	LS mean	se	LS mean	se	LS mean	se	LS mean	se	*P*
Log LCβC	−0·66	0·07	−0·14	0·1	−0·78	0·07	−0·93	0·11	−1·07	0·1	<0·001
Log FABP4	1·41	0·04	1·37	0·03	1·52	0·05	1·49	0·37	−0·13	0·05	0·025
Log FABP5	0·7	0·06	0·7	0·05	0·74	0·06	0·76	0·05	−0·01	0·06	0·9
Log HOMA-IR†	1·27	0·1	1·2	0·1	1·34	0·1	1·43	0·1	−0·23	0·1	0·014
Log leptin	1·22	0·06	1·2	0·08	1·26	0·06	1·34	0·06	0·54	0·03	0·297
Log adiponectin	3·95	0·05	4·07	0·05	4·04	0·05	4·04	0·06	0·03	0·02	0·192

LCβC, lipid-corrected β-carotene; FABP, fatty acid binding protein; HOMA-IR, homeostasis model assessment-insulin resistance.

* Adjusted for percentage change in body weight.

† Log HOMA-IR data have been previously reported^(^^19^^)^.

Subjects were classified lean if BMI was ≤85th percentile and OW if BMI was >85th percentile. By design, significant differences between the lean and OW groups exist at baseline in terms of adiposity including BMI *Z*-score and abdominal fat mass (*P* ≤ 0·05; Table 1). Pill count data were available for 88 % of all supplement bottles dispensed. At 3 months, 60 % of the lean and 55 % of the OW subjects had taken >75 % of the active supplement capsules, and at 6 months, 80 % of the lean and 50 % of the OW children had >75 % compliance. However, the groups were not statistically different.

### Correlations

**Fig. 1.**
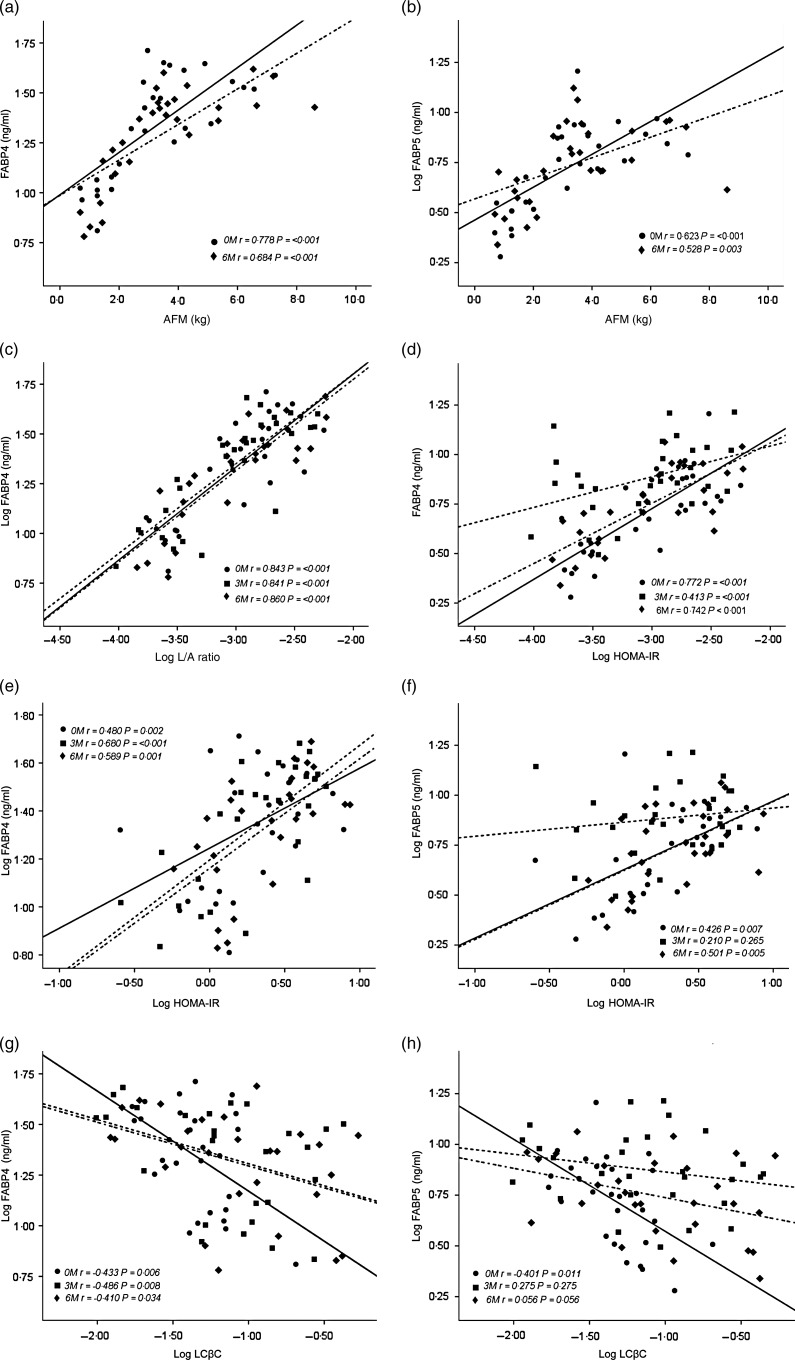
Pearson's correlations between fatty acid binding protein (FABP) 4 and FABP5 and abdominal fat mass (AFM) in kg (a, b), leptin:adiponectin ratio (L/A) (c, d), homeostasis model assessment-insulin resistance (HOMA-IR) (e, f) and lipid-corrected β-carotene (LCβC) (g, h) at baseline (––; 0M; ●), 3 months (- - - -; 3M; ■) and 6 months (–. –; 6M; ♦).

FABP4 and FABP5 were higher in the OW *v.* lean boys (Table 1) and directly correlated with abdominal fat mass and leptin:adiponectin ratio at baseline and throughout the study (Fig. 1(a)–(d)); FABP4 (*P* = 0·002) and FABP5 (*P* = 0·007) were also directly correlated with log HOMA-IR at baseline, but FABP5 lost its correlation at 3 months post-intervention (*P* = 0·265) (Fig. 1(e) and (f)). FABP4 (*r* 0·823; *P* ≤ 0·001) and FABP5 (*r* 0·699; *P* ≤ 0·001) were directly correlated with leptin. FABP4 (*r* −0·439; *P* = 0·015) was negatively correlated with adiponectin at baseline and maintained its correlation throughout the study time points. FABP5 (*r* −0·370; *P* = 0·044) was negatively correlated with adiponectin at baseline, but lost its correlation at the 3- and 6-month time points (data not shown). In contrast, FABP4 (*P* = 0·006) and FABP5 (*P* = 0·011) had a negative correlation with lipid-corrected β-carotene at baseline, but FABP5 lost its correlation at 3 months (Fig. 1(g) and (h)). FABP4 (*P* = 0·014) and FABP5 (*P* = 0·005) were directly correlated with high-sensitivity C-reactive protein, but only FABP5 was directly correlated with IL-6 (*P* = 0·015) at baseline. In multiple regression analyses, BMI *Z*-score explained 66 % of the variance for FABP4 and 47 % of the variance for FABP5. When adiponectin was added to the model, 75 % of the variance was explained by FABP4 and 49 % by FABP5 (*P* < 0·001) for all. No collinearity violations were observed between variables.

### Treatment effects

**Fig. 2.**
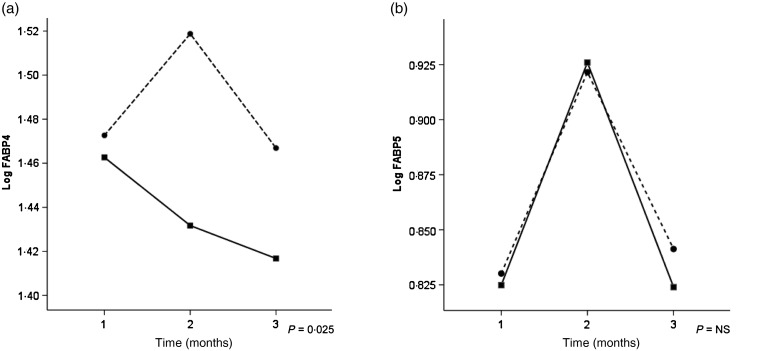
Fatty acid binding protein (FABP) 4 (a) and FABP5 (b) changes over time (months) between fruit and vegetable juice concentrate-supplemented boys (–■–) and those receiving placebo (--●--) in the overweight group. Values are least squared means.

FVJC supplementation substantially reduced FABP4 (*P* = 0·025) when corrected for percentage change in abdominal fat mass, as opposed to FABP5, which showed no significant treatment effect (*P* = 0·231) when compared with placebo (Table 2 and Fig. 2). Wilcoxon signed-ranks tests determined inverse correlations between the percentage change in log lipid-corrected β-carotene (*Z* = −2·77; *P* = 0·005) and FABP4 (*Z* = −2·128; *P* = 0·033) but not for FABP5 (*Z* = −0·896; *P* = 0·370).

There were no significant treatment effects between groups for BMI *Z*-score or total body fat. There was a significant treatment effect on the percentage change in abdominal fat mass (kg) measured by dual-energy X-ray absorptiometry for the entire cohort at 6 months, showing that the placebo group had a 11·2 (95 % CI 4·16, 18·23) % increase, as opposed to a 1·47 (CI −8·31, 5·37) % decrease in the supplement group (*P* = 0·029).

## Discussion

In this *post hoc* analysis study, we report not only the effects of childhood adiposity and related metabolic alterations on FABP4 and FABP5 in prepubertal boys but also the outcome of a 6-month intervention with a FVJC supplement on the concentration of the FABP. The data demonstrated higher concentrations of both FABP4 and FABP5 in the OW when compared with the normal-weight boys. While both FABP4 and FABP5 were closely associated with insulin resistance and measures of adiposity, only FABP4 showed a decrease in concentration in response to the 6-month intervention with the FVJC supplement.

The higher levels of FABP4 in OW boys when compared with the levels of their lean counterparts that were observed in our study are similar to previous reports in children^(^^12^^,^^13^^,^^15^^,^^16^^,^^18^^)^ and adults^(^^3^^,^^9^^–^^11^^,^^14^^,^^17^^,^^24^^)^, but the increased concentration of FABP5 has not been previously reported. Both FABP4 and FABP5 are altered in childhood obesity and showed direct associations with markers of insulin resistance including AIR and HOMA-IR, as well as the leptin:adiponectin ratio, which also has been suggested as an indicator of insulin resistance^(^^25^^)^. Our data in non-diabetic prepubertal boys agree with previous observations in adults that FABP4 plays an important role in the AIR^(^^17^^)^. Further, the strong correlations between both FABP and abdominal adiposity observed in the present study suggest that FABP may contribute to the obesity phenotype in boys.

β-Carotene is the main carotenoid in human sera and has been proposed as a useful indicator of fruit and vegetable consumption^(^^26^^,^^27^^)^. While there was an inverse relationship between both FABP and baseline β-carotene concentration, only the magnitude of decrease in FABP4, and the corresponding increase in β-carotene concentration were correlated. The FVJC consumption resulted in a significant increase in β-carotene and a decrease in abdominal fat mass, but only the concentration of FABP4 and not FABP5 was reduced. FABP4 is highly expressed in adipocytes, whereas FABP5 is predominantly epidermal and constitutes only a minor fraction of FABP in the adipocytes (the amount being almost 100-fold lower than that of FABP4)^(^^28^^)^. Although metabolically complementary to FABP4, FABP5 did not respond to the FVJC supplementation in a similar fashion, suggesting that the two FABP have a differential response to an external stimulus such as polyphenolic supplementation. It is not known if a similar pattern of response holds for other interventions such as exercise and/or other nutritional/pharmacological therapies.

The underlying mechanisms leading to the specific reduction in FABP4 levels in response to the FVJC supplementation are not apparent from the present study. Responses to dietary supplementation with these compounds can be quite variable between individuals^(^^29^^)^ and may have influenced the results of this study. Genome-wide studies have associated common genetic polymorphisms in the β-carotene oxygenase 1 (*BCO1*) gene with the obese phenotype in African-American adults^(^^30^^)^. *BCO1* is the gene that encodes an enzyme that is expressed in the intestine and converts provitamin A carotenoids to retinaldehyde and all-*trans*-retinoic acid; both can stimulate retinoic acid receptors and reduce PPARγ activity. The reduction in PPARγ activity may lead to a decrease in the expression of PPARγ target genes such as FABP4 with subsequent decreases in adipocyte lipid content, as has been previously reported in mature adipocytes^(^^31^^)^ and mouse models^(^^2^^,^^32^^,^^33^^)^. Other anti-adipogenic cooperative or alternative mechanisms may involve flavonoid-mediated inhibition of Akt activation and GSK3β phosphorylation, which induces down-regulation of lipid accumulation and lipid-metabolising gene expression, ultimately inhibiting adipocyte differentiation^(^^34^^,^^35^^)^. Mice with deletion of both FABP4 and FABP5 have strong protection from diet-induced obesity, insulin resistance, type 2 diabetes and fatty liver disease^(^^5^^)^. A small-molecule dual inhibitor of FABP4/5, when chronically administered to diet-induced obese mice, was recently reported to reduce plasma TAG and NEFA levels, without inducing significant changes in insulin sensitivity^(^^36^^)^. Pharmacological agents that modify FABP function may provide tissue-specific or cell-type-specific control of lipid signalling pathways, inflammatory responses and metabolic regulation, potentially providing a new class of drugs for diseases such as obesity, diabetes and atherosclerosis^(^^37^^)^. Emerging data from human studies suggest that inhibiting the function of FABP4 may be a potential mechanism for the prevention of metabolic diseases like type 2 diabetes and atherosclerosis^(^^37^^,^^38^^)^. In the Nurses' Health Study and the Health Professionals Follow-up Study, a functional variant T87C polymorphism in the FABP4 promoter resulted in reduced adipose FABP4 messenger ribonucleic acid expression and was associated with reduced risk for metabolic dyslipidaemia, type 2 diabetes and coronary atherosclerosis^(^^9^^)^. In this context, the lowering of FABP4 in our study via low-dose supplementation with FVJC and NC is important. Using the Penn Diabetes Heart Study, cross-sectional analysis of FABP4 and FABP5 levels had an additive, independent association with the metabolic syndrome and inflammatory markers, but only FABP4 showed a specific association with the presence of coronary artery Ca^(^^39^^)^. Previous studies based on exercise interventions in obese children and adults have shown substantial deceases in FABP4 levels^(^^11^^,^^35^^)^. In obese children undergoing a 1-year weight-reduction programme, changes in FABP4 concentrations correlated significantly with percentage body fat and leptin, but the decrease in FABP4 levels was limited only to those children who lost substantial amounts of weight^(^^12^^)^. These results are similar to our study. A larger study in prepubertal children also showed that FABP4 was significantly associated with changes in both weight and waist circumference over 3 years of follow-up, and higher concentrations at baseline predicted the development of the metabolic syndrome^(^^16^^)^; these results are similar to those reported in adults^(^^40^^)^. Another study in children found that FABP4 predicted metabolic syndrome components with almost 68 % accuracy independent of BMI status and that a weight-reduction programme with diet and exercise was effective in reducing FABP4, BMI percentile, waist circumference, TAG and cholesterol^(^^15^^)^. The beneficial improvements in FABP4, insulin resistance, TAG levels, abdominal fat, and the differential regulation of FABP with NC and the FVJC supplementation suggest that carotenoids, along with other polyphenols, may play a role in the regulation of abdominal adiposity, similar to that reported in animal models^(^^32^^,^^41^^)^, and warrant further exploration in the paediatric population^(^^19^^,^^42^^)^.

Strengths of the study include the randomised double-blind, placebo-controlled prospective nature of the intervention and the inclusion of both lean and obese subjects. The limitations of the study include the relatively small sample size; lower than expected rates of compliance; lack of inclusion of both sexes in the study cohort; and the use of surrogate markers for insulin resistance, β-cell function and abdominal adiposity. We also did not measure all-*trans*-retinoic acid; however, a previous intervention study using carrot juice, which is rich in β-carotene, reported a doubling of plasma all-*trans*-retinoic acid levels without significant increase in serum retinol^(^^43^^)^. The data were not adjusted for compliance rates because there were no significant differences between groups. Further, β-carotene represents only one of several polyphenolic compounds present in the supplement^(^^21^^)^, and this precludes us from drawing definitive conclusions about ascribing a unique role to β-carotene as solely responsible for the observed effects in the present study.

In conclusion, in this *post hoc* analysis, we report higher levels of both FABP4 and FABP5 in OW *v.* lean prepubertal boys and show a reduction in circulating FABP4 along with beneficial improvements in insulin sensitivity and abdominal adiposity in the children supplemented with the FVJC for a sustained period of 6 months. Although the mechanisms remain unclear, given the strong relationship between FABP4 and indicators of adiposity and metabolic dysfunction and the beneficial effect of the supplementation, future studies are warranted to corroborate these findings.
